# Influence of operator’s experience on complications of root canal treatment using contemporary techniques: a retrospective study

**DOI:** 10.1186/s12903-024-03876-9

**Published:** 2024-01-17

**Authors:** Tongfei Shao, Rui Guan, Chen Zhang, Benxiang Hou

**Affiliations:** 1 Department of Endodontics, School of Stomatology, Capital Medical University, Beijing, China; 2 Center for Microscope Enhanced Dentistry, School of Stomatology, Capital Medical University, No. 28 Xin Rong Street, Daxing District, Beijing, 100162 China

**Keywords:** Root canal treatment, Complications, Operator experiences, Contemporary techniques

## Abstract

**Background:**

Endodontic treatment has benefited from the development of new techniques and equipment. Few clinical studies have been published on the complications associated with root canal preparations performed by doctors with different working experiences using contemporary techniques. This study aimed to analyze the complications of endodontic treatment performed by residents and endodontic specialists in a teaching stomatology hospital using contemporary techniques.

**Methods:**

Cases of root canal treatment (RCT) and non-surgical root canal retreatment (ReRCT) performed by residents with 1–3 years of experience and endodontic specialists with 5–7 years of experience were collected from the electronic medical system of the Department of Endodontics, Beijing Stomatology Hospital, from September 1, 2020 to August 31, 2021. The cases were examined in terms of patient age, sex, type of tooth, diagnosis, treatment modality (RCT or ReRCT), number of appointments, whether an operating microscope was used, presence of ledges, canal transportation, perforations, missed canals, separated instruments, flare-ups and clinical incidence of second mesiobuccal (MB2) root canal in the maxillary molars.

**Results:**

In total, 859 teeth from 820 patients were included in the analysis. The overall incidence of complications in the resident group was significantly higher than that in the specialist group. More ledges and flare-ups were observed in the resident group (*p* < 0.05). The clinical incidence of MB2 was significantly higher in the specialist group (*p* < 0.05). There were no significant differences in root canal transportation, perforation, or instrument separation between the two groups (*p* < 0.05). Multivariate analysis showed that the incidence of root canal preparation complications was related to operator experience, tooth type and treatment modality.

**Conclusions:**

Technical advancements could reduce the effect of working experience on RCT complications between residents and endodontic specialists in a teaching stomatology hospital.

## Background

The preparation of root canals is one of the most important procedures in endodontic treatment, shaping root canals, and preparing them for root canal filling. Complications may occur during root canal preparation due to the complexity and irregularity of the root canal system. Root canal complications, or “catastrophic errors,” which include ledges, canal transportation, perforations, separated instruments, missed canals, and flare ups, might ultimately lead to treatment failure [1–6].

Previous studies have reported that ledges, apical transportation, and foramen perforations are commonly observed during root canal instrumentation in undergraduate students using stainless-steel K-files [7, 8]. Better canal preparations were achieved using rotary nickel-titanium (NiTi) instruments than with manual files [9, 10]. Experience has been found to influence ledges, apical transportation, foramen perforations, the location of additional canals and the prevalence of postoperative pain [1, 2, 11, 12]. Most of these studies were published between 2000 and 2010 and used stainless-steel K-files or traditional rotary NiTi instruments [1, 2, 7–12]. However, two studies published in 2014 and 2017 show that the introduction of new technologies could improve the technical standards of undergraduate root filling and operator experience does not influence shaping ability in a single-file reciprocating motion system, which indicates that technical advancement could reduce the effect of working experience on root canal treatment (RCT) in some ways [13, 14].

In recent years, endodontic treatment has benefited from the development of new techniques and equipment. The use of operating microscopes and ultrasonic instruments has improved the efficiency and outcomes of endodontic treatment [15, 16]. With the intention of reducing instrument separation and improving the shaping ability of root canal preparation, new processing technologies and thermal treatments have improved the physical and mechanical properties of NiTi instruments, resulting in better torsional resistance, fatigue lifetime, and flexibility [17–19]. To our knowledge, few clinical studies on the complications of RCTs performed by doctors with different working experiences using contemporary techniques have been published to date. Whether technical advancement could reduce the effect of working experience on RCTs complications and whether there is a greater risk of serious complications in a teaching stomatology hospital require further study. The aim of this study was to analyze the complications of RCTs performed by residents and endodontic specialists at the Beijing Stomatology Hospital using contemporary techniques.

## Methods

### Ethical approval and informed consent

This study was approved by the Research Ethics Committee of Beijing Stomatology Hospital, Capital Medical University (reference no. CMUSH-IRB-KJ-PJ-2023-53), and was in compliance with the Helsinki Declaration, and that each subject in the project signed a detailed informed consent form.

### Case selection

Cases of RCTs and non-surgical root canal retreatment (ReRCT) performed by residents with 1–3 years of working experience (Group R) and endodontic specialists with 5–7 years of working experience (Group S) were collected from the electronic medical system of the Department of Endodontics, Beijing Stomatology Hospital, Capital Medical University, Beijing, China, from September 1, 2020 to August 31, 2021. Cases lacking complete dental records or preoperative or postoperative radiographs, cases involving the third molar, and those in which the apical foramen was not fully completed and needed an apical barrier were excluded from the study.

### Endodontic therapy procedure

For each case, the following information was recorded from the electronic medical system: patient age, sex, date of treatment, type of tooth, diagnosis, treatment modality (RCT or ReRCT), number of appointments, whether an operating microscope was used, clinical incidence of the MB_2_ root canal in the maxillary first and second molars, and occurrence of flare-ups.

The procedure for the RCT is briefly described as follows. Conventional straight-line access preparation was performed after rubber-dam application to ensure isolation. An operating microscope was used to locate the root canal, and ultrasonic instruments were used to gain access to the canal openings. Root canal preparations were performed using the crown-down instrumentation technique with M3-Pro (United Dental, Shanghai, China) rotary NiTi files manufactured using a controlled memory (CM) wire. The preparation was performed according to the manufacturer’s instructions. The preparation sequence was a 17/08 M3-Pro file to enlarge the coronal two-thirds of the canal, followed by #8, #10, #15 C-file and 19/02, 20/04, 25/04, 25/06 M3-Pro files to the full working length; a 35/04 file may be used if necessary. The working length was determined using an electronic apical locator (Raypex 6; VDW, Munich, Germany) and confirmed using digital radiographs, if necessary. For ReRCTs, previous obturation materials and root canal obstructions were removed using hand files, NiTi files, and ultrasonic instruments under the magnification and lighting of the operating microscope. According to the root canal anatomy and infection severity, the final apical file of the ReRCTs was at least 25/06 or 35/04 in curved root canals, and 40/05 in anterior teeth. Root canals were irrigated with 2.5% sodium hypochlorite solution. Therapy was completed during single or multiple treatment visits. In the latter case, calcium hydroxide paste was placed in the canal between appointments. Root canal obturation was performed using the warm vertical compaction technique. Cone-beam computed tomography (CBCT) was performed during the treatment procedure, if necessary.

### Evaluation of complications of RCT

Two endodontists with > 7 years of experience evaluated the preoperative, intraoperative, and postoperative radiographs, as well as any progress notes that mentioned one of the procedural errors. In case of controversy, a highly experienced endodontist was consulted to evaluate the radiographs and a final decision was made.

The examiners used the following criteria to evaluate RCT complications by reading radiographs [2, 7, 20, 21]. Ledges were identified when the root canal filling deviated from the original canal shape or when the root filling was more than 2 mm short of the radiographic apex. Root canal transportation was identified when the root canal filling deviated inside or outside the curvature of the root canal, or when the root apex was overcut so that the original position was to be changed. Root perforation was identified as communication between the root canal space and the external root surface. Instrument separation was identified when a radiopaque fractured instrument segment was detected in the root canal or when it extended into the periapical area. Missed canals were identified when the obturation material was positioned asymmetrically along the long axis of the root.

### Statistical analysis

Statistical analyses were performed using SPSS for Windows, version 26.0 (IBM, Armonk, NY). The demographic data were presented as frequencies and proportions for categorical variables and as medians and interquartile ranges for continuous variables. Categorical variables were compared using the chi-square test or Fisher’s exact test for equal proportions. Dichotomous variables were presented as percentages and were compared using Fisher’s exact test. Univariate and multivariate analyses were used to evaluate joint associations among various factors using logistic regression models. All the statistical tests were two-tailed and interpreted at a 5% significance level.

## Results

A total of 881 teeth from 842 patients were included in this analysis. After excluding 2 cases with a third molar and 20 cases that required an apical barrier, a total of 859 teeth from 820 patients were included (401 cases in Group S and 458 cases in Group R).

### Basic information of patients and teeth

The basic information of the two groups was shown in Table 1. There were no significant differences in sex between the two groups. The patients in Group S were significantly older than those in Group R. Specialists treated significantly more molars, more teeth that needed ReRCT, and more teeth with apical periodontitis than residents. There were significantly fewer appointments in Group S than in Group R. In the process of root canal preparation and obturation, the rates of using operating microscope were significantly higher in Group S than in Group R.

**Table 1:** The basic information of the two groups

	Specialists (*n* = 401)	Residents (*n* = 458)	p
Age	37(28, 50)	33(27, 42)	<0.001
Sex (female)	242(60.3%)	297(65.0%)	0.161
Tooth with apical periodontitis	146(36.4%)	106(23.1%)	<0.001
Type of tooth			
Anterior tooth and premolar	165(41.1%)	242(52.8%)	0.001
Molar	236(58.9%)	216(47.2%)	0.001
Root canal retreatment	95(23.7%)	49(10.7%)	<0.001
Number of appointments	2.3 ± 0.6	2.9 ± 0.9	*P* = 0.002
Using operating microscope during root canal preparation	396(98.8%)	207(45.2%)	<0.001
Using operating microscope during root canal obturation	397(99.0%)	239(52.2%)	<0.001

### Complications of RCT

The overall incidence of complications was significantly higher in Group R than in Group S. More ledges and flare-ups were observed in Group R. There were no significant differences in root canal transportation, root perforation, or instrument separation between the two groups (Table 2).

**Table 2:** Frequency of complications in the two groups

	specialists *n* = 401	residents *n* = 458	p
Total Complications	32(8.0%)	72(15.7%)	0.001
Flare up	8(2.0%)	22(4.8%)	0.025
Ledge	16(4.0%)	37(8.1%)	0.013
Root canal transportation	1(0.2%)	5(1.1%)	0.139
Root perforation	1(0.2%)	4(0.9%)	0.230
Missed canal	2(0.5%)	5(1.1%)	0.335
Instrument separation	4(1.0%)	6(1.3%)	0.670

### Findings of MB2

**Fig. 1:**
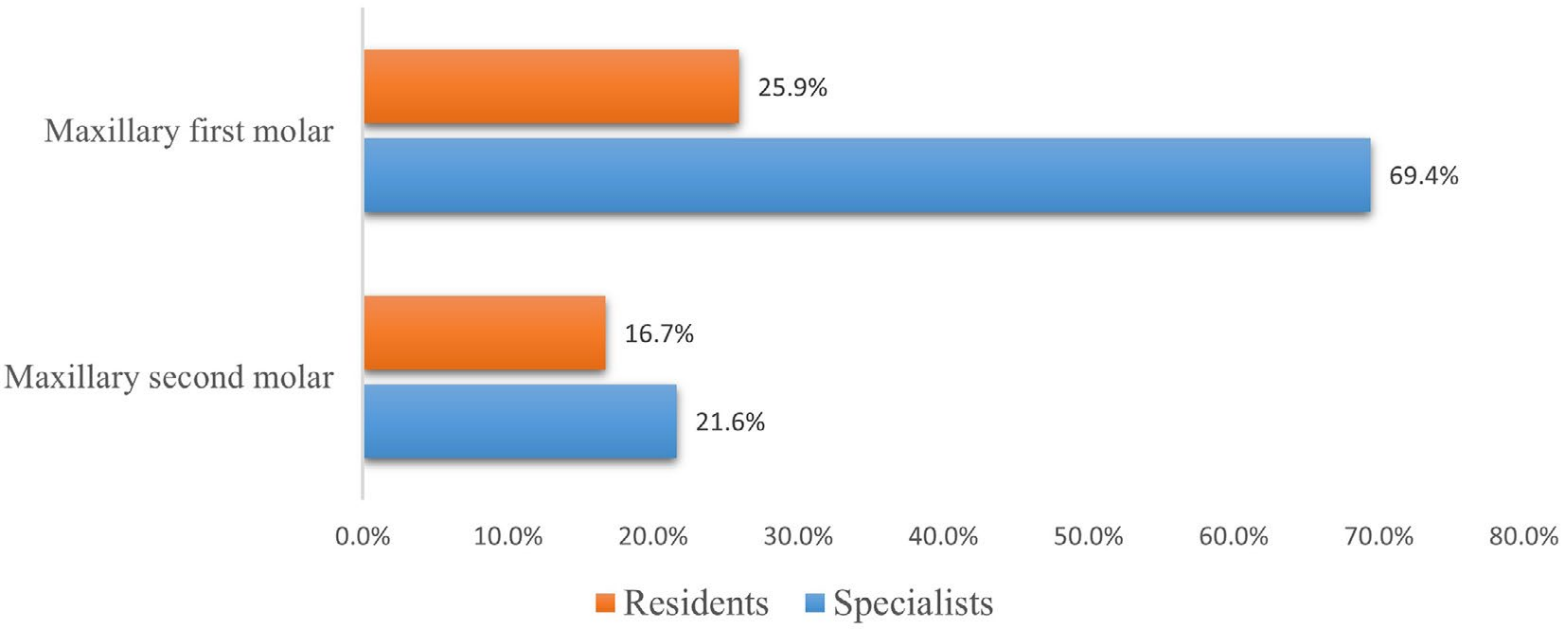
The clinical incidence of MB2 in the maxillary first and second molar in the two groups

There were 58 and 36 maxillary first and second molars in Group R and 62 and 51 in Group S, respectively. The clinical incidence of MB2 canals in the maxillary first and second molars was significantly higher in Group S than in Group R (Fig. 1).

### Univariate and multivariate analysis of complications

Univariate analysis revealed that the incidence of complications was related to operator experience and tooth type. After adjusting for multivariate analysis, complications were found to be associated with operator experience, tooth type, and treatment modality (Table 3).

**Table 3:** Univariate and multivariate analysis of complications

	Univariate analysis		Multivariate analysis	
	OR [95% CI]	p	OR [95% CI]	p
Residents	2.151[1.385–3.340]	0.001	2.303[1.355–3.913]	0.002
Molar	2.010[1.303–3.102]	0.002	2.359[1.498–3.715]	<0.001
Root canal retreatment	1.488[0.900- 2.458]	0.121	1.953[1.148–3.324]	0.014
Age	0.986[0.971–1.002]	0.091	0.991[0.975–1.008]	0.312
Using operating microscope during root canal preparation	0.805[0.506–1.282]	0.362	0.752[0.449–1.257]	0.277

## Discussion

In this retrospective study, initial endodontic treatment and non-surgical endodontic retreatment were performed by residents with 1–3 years of working experience and endodontic specialists with 5–7 years of working experience in the Department of Endodontics, Beijing Stomatology Hospital, Capital Medical University. As a stomatology hospital, a certain number of cases were referred from general hospitals, community hospitals, or private clinics that had calcified or curved root canals or failed initial endodontic treatment that required non-surgical retreatment. Beijing Stomatology Hospital is also a teaching hospital that undertakes many teaching tasks and trains medical students at different stages, including undergraduates, postgraduates, and residents. Therefore, it is worth investigating whether there is a higher risk of serious complications. Previous clinical studies have reported that students produce more complications. However, these clinical studies were conducted before many modern endodontic techniques became available, using Gates–Glidden burs and stainless-steel hand files for root canal preparation between 2000 and 2010 [1, 2, 7, 8]. In our study, endodontic treatment was performed using modern techniques, such as ultrasonic instruments, CM wire NiTi file systems, operating microscopes and CBCT scanning. As residents have a few years of clinical experience, they can perform certain complicated endodontic treatment procedures independently; therefore, we chose residents and young specialists as research subjects. The present study was performed to determine whether there is a greater risk of serious root canal complications when performed by residents and young endodontic specialists using modern endodontic techniques in a teaching stomatology hospital.

The root canals of molar teeth are more curved and narrower than those of premolar and anterior teeth. Older adults are more likely to have calcified canals due to aging-related changes, which presents a practical challenge to clinicians during RCTs [22]. In addition, the healing rate of teeth with lesions was significantly lower than that of teeth without lesions, requiring stricter treatment standards [1]. Moreover, several challenges are faced during retreatment, including the removal of the previous obturation material, correction of procedural errors generated during the previous treatment, such as ledges, root canal transportation or perforation, locating missed canals, eliminating infected pulp and therapy-resistant bacteria. Our results indicated that more complex cases were treated by endodontic specialists than residents, which consisted of more molars (58.9% vs. 47.2%, *p* = 0.001), more teeth with periapical lesions (36.4% vs. 23.1%, *p* < 0.001), and more ReRCTs (23.7% vs. 10.7%, *p* < 0.001). The reason may be that more complex cases were referred to specialists, or that patients had previously registered with a doctor at the senior level according to the difficulty of treatment.

Although specialists treat more difficult cases, they actually needed fewer appointments to complete treatment than residents (2.3 ± 0.6 vs. 2.9 ± 0.9, *p* = 0.002). These results are similar to those of previous studies [23, 24]. This may be because specialists are more knowledgeable, professional, and skillful. When dealing with some basic steps, such as access to the pulp chamber, removal of filling material or the negotiation of calcified root canals by the #8 and #10 C-files can still be obstacles that differentiate the number of appointments used by residents and specialists. In addition, although the microscope requires extensive prior training, it can be an interesting tool for endodontic specialists since the use of magnification during treatment can make the procedure faster [25]. In the Department of Endodontics, Beijing Stomatology Hospital, every dental unit is equipped with an operating microscope, including students’ consultation room and specialists’ consultation room. Teachers also trained students to use operating microscope from the undergraduate stage. However, in the present study, the rate of operating microscope use during root canal preparation by residents was only 45.2%, which was significantly lower than that used by specialists (98.8%). The specialists were more accustomed to using an operating microscope as a conventional procedure during treatment with its appropriate magnification and illumination, for the operating microscope enables endodontists to resolve treatment challenges previously unrecognized or untreatable, which has been encouraged by the AAE to improve endodontic outcomes [26].On the one hand, cases performed by residents were not as complicated as those performed by specialists; on the other hand, residents may be unproficient in using an operating microscope, and unaware of the difficulty of certain cases, such as identifying the MB2 root canal of the maxillary molar. As such, they chose not to use the operating microscope in some cases. Therefore, with their professional experience and use of operating microscopes as conventional procedures, specialists are more efficient in their treatment. What’s more, training in microscopes has to be reinforced in residents’education.

More ledges were observed in the resident group. Residents were not as skilled or knowledgeable as specialists and were not familiar with the anatomical morphology of the root canal. They may not be aware of pre-curved hand files when negotiating curved canals. However, although residents produced more ledges, the rate of the resident group was only 8.1% in our study, which was lower than that reported in previous studies [2, 7, 8]. This may be because a contemporary treatment procedure was followed, which represents the current standard of endodontic therapy in our study, including the use of operating microscopes and ultrasonic instruments to achieve glide path, and CM-wire rotary instruments to prepare root canals.

In addition, more flare-ups were observed in the resident group. Flare-ups are a multivariate problem in clinical practice, but one of the principal causes is inflammation caused by the extrusion of contaminated debris into periradicular tissues [27]. This may be attributable to the dexterity and skill of the operators. Residents have a poorer ability to control the working length during treatment than specialists; therefore, instruments may extend beyond the apical foramen. Once flare-ups appeared, residents needed additional inter-appointment medications to lessen symptoms, which led to more treatment appointments. However, the main concern of postoperative pain studies is the subjective evaluation of pain levels. In our study, severe percussion pain and swollen gums were used to identify flare-ups, which could reduce the interference of subjective consciousness.

We examined several factors, including age, tooth type, treatment modality, use of operating microscope during root canal preparation and operator experience, to assess significant outcome predictors. The results indicated that the type of tooth and operator experience significantly affected the outcome according to the univariate and multivariate logistic regression models, and the treatment modality significantly affected the outcome according to the multivariate logistic regression models. Our results were similar with those of previous studies reporting that the occurrence of complications was affected by the type of tooth and the root canal curvature [2, 20]. When dealing with ReRCTs, correcting procedural errors, such as ledges, root canal transportation, and perforation, has been a major challenge.

Root canal transportation and perforation were slightly more frequent in the resident group, but with no significant differences between the two groups. This might be attributable to the improved torsional resistance, fatigue lifetime, and flexibility of the CM-wire NiTi instruments, as several studies have highlighted their benefits [17–19, 28]. Study shows less proficient operators were associated with a higher incidence of file separation, as the incidences of instrument separation in the present study were 1% and 1.3% in the specialist group and resident group respectively, but with no significant differences either [29]. It seems that technical advancements in instrument could reduce the effect of working experience on root canal preparation. However, another study shows the incidence of CM wire instrument separation was relatively high, with fracture rates of 8.5% and 3.69% considering the number of teeth and canals treated [30]. The CM wire NiTi system used in the above study was developed for the purpose of making more conservative biomechanical preparations by means of less wear on the peri-cervical dentin, which lacks a file to enlarge the coronal two-thirds of the canal. Besides, operating microscope and ultrasonic instruments were not used either. Therefore, an effective procedure for creating straight-line access and enlarging the coronal of the canal was deficient, which leading to the increasing treatment difficulty of apical third of the canal. That may be the main reason for the high rate of instrument separation in the apical third of molars in their study.

In the present study, the clinical incidences of MB2 canals in the maxillary first and second molars identified by residents were 25.9% and 16.7%, respectively, which were significantly lower than that identified by specialists (69.4% and 21.6%, respectively). Our results by specialists were in line with previous studies that showed that the incidence of MB2 canals was 62–69% in the laboratory phase [31, 32], 71% and 77% by experienced endodontists [33, 34]. However, the incidence of MB2 canals among residents was relatively low. Research has confirmed that operating microscopes and operator experience affect the location of additional canals in the MB root of maxillary molars [11, 15]. In our study, the rate of operating microscope use during root canal preparation by residents was only 45.2%, which was significantly lower than that of specialists (98.8%). Therefore, whether residents were aware of looking for extra canals using operating microscopes and ultrasonic instruments was most likely to account for the findings of MB2 canals.

## Conclusions

Despite the limitations of this retrospective study, the results indicated that more complex cases were referred and treated by endodontic specialists. There is not a greater risk of serious root canal complications performed by residents using modern endodontic techniques in a teaching stomatology hospital. Technical advancements could reduce the effect of working experience on RCT complications between residents and endodontic specialists. A special training modality may be useful in designing future clinical trials to draw definitive conclusions on the effects of reducing the complications of RCTs.

## Data Availability

The datasets used and/or analyzed during the current study are available from the corresponding author on reasonable request.

## Abbreviations

RCT: root canal treatment
NiTi: nickel-titanium
ReRCT: root canal retreatment
CM: controlled memory
CBCT: cone-beam computed tomography
